# Lymphangioma Circumscriptum on the Buccal Mucosa: A Case Report of a Rare Entity

**DOI:** 10.7759/cureus.70466

**Published:** 2024-09-29

**Authors:** Ramachandra Reddy Gowda Venkatesha, Ezhilarasi Arumugam Venkatachalam Sargurunathan, Karthik Rajaram Mohan, Nandhinidevi Govindharaju, Mahajan Eshwar Tanaji

**Affiliations:** 1 Oral Medicine and Radiology, Vinayaka Mission's Sankarachariyar Dental College, Vinayaka Mission's Research Foundation (Deemed to Be University), Salem, IND; 2 Oral and Maxillofacial Pathology, Vinayaka Mission's Sankarachariyar Dental College, Vinayaka Mission's Research Foundation (Deemed to Be University), Salem, IND; 3 Oral and Maxillofacial Surgery, Vinayaka Mission's Sankarachariyar Dental College, Vinayaka Mission's Research Foundation (Deemed to Be University), Salem, IND

**Keywords:** benign lymphangioma, buccal mucosa alterations, excision biopsy, papules, reactive hyperplasia

## Abstract

Hamartomatous lymphatic channel proliferation causes lymphangiomas or microcystic lymphatic malformations (MLM). They are most commonly found in the head and neck, with oral occurrences a rarity. In this case, a 34-year-old woman presented with lymphangioma circumscriptum on the buccal mucosa, a condition that typically causes asymptomatic pebbly papules. This article focuses on the clinical features of this unique case, shedding light on the treatment modalities and histological features.

## Introduction

Lymphangioma circumscriptum (LC) is a specific type of lymphangioma, a broader term used to describe a group of conditions involving malformations of the lymphatic vessels [1]. Lymphangiomas are benign lymphatic system anomalies that can appear on the skin and mucous membranes. They are uncommon and can cause no symptoms [2]. A few occurrences of squamous cell carcinoma, verruciform xanthoma, and lymphangiosarcoma inside lymphangiomas have been documented [2]. The term "circumscriptum" refers to the fact that the lesion in this type of lymphangioma is usually well-circumscribed and has a clear boundary. A very unusual lymphatic abnormality, LC, is located on the outer layer of the skin. These lesions can be present at birth or developed over time. Lesions of LC are clinically identified by "frog-spawn" clusters with thin-walled, transparent vesicles. The pathogenesis of LC, which was initially described by Whimster in 1970, is thought to involve deep lymphatic cisterns that compress on superficial lymphatics, leading to saccular dilatations in the epidermis and, ultimately, blisters on the skin [3]. In most cases, there is no preference based on sex; however, a male-to-female ratio of 2:1 is observed in cases of small-sized lymphangiomas (<1 cm) of the alveolar mandible ridge. According to the literature, lymphangiomas occur between 1.2 and 2.8 times per 1000 infants [4]. Nwoga reported the prevalence of lymphangioma in Enugu among the Nigerian population was about 6.4% [5].

The exact cause of LC is unknown, but it's thought to be related to abnormal development of the lymphatic vessels during embryogenesis. Some possible risk factors for congenital lymphangiomas include genetic predisposition, acquired lymphangiomas resulting from trauma or injury to the affected area, and infection or inflammation. Fibroblasts surrounding lymphatics and lymphatic endothelial cells generate excessive amphiregulin, which causes congenital cystic lymphangioma - the theories implicated in the pathogenesis of lymphangioma [6]. Congenital lymphatic malformations can occur from major lymphatic channels being sequestered, blocked primitive lymphatic channels unable to join main lymphatic arteries or veins during embryogenesis. The second theory proposes that the primitive lymphatic sac does not reach the venous system, and hence, lymphatic pathways cannot empty into the veins, causing lymph ectasia and stasis. The latest theory suggests that lymphangiomas exhibit increased expression of vascular endothelial growth factor ligand (VEGF-C) and its receptor (VEGF-R3), while showing reduced levels of pigment epithelium-derived factor and thrombospondin-1, both of which are inhibitors of angiogenesis. Trauma, cancer, surgery, and radiation therapy can alter lymphatic outflow, causing acquired lymphangiomas [7].

When LC occurs on the buccal mucosa, symptoms may include a painless, soft, compressible mass or swelling on the inner cheek. The mass may have a bluish or translucent colour due to the presence of lymphatic fluid. In some cases, the lesion may bleed or ulcerate. If the lesion is large enough, it can interfere with oral function, leading to difficulties in speech (dysarthria), eating, or swallowing (dysphagia) [7]. Cervical lymphangiomas occur in the neck as masses, which extend a size of up to 10 cm in diameter and cause pain or compression of adjacent vital structures. Lymphangiomas are histopathologically classified as cystic lymphangiomas (cystic hygromas), lymphangioma simplex (capillary lymphangioma), and cavernous lymphangioma [8]. Radiographically, lymphangiomas can be classified as macrocystic (>2 cc in volume), microcystic (<2 cc), or mixed (≥50% macrocystic component) [8].

Diagnosis is typically based on clinical examination and medical history, along with imaging studies, such as ultrasound, contrast-enhanced CT, MRI scans, and biopsy.

## Case presentation

**Figure 1:**
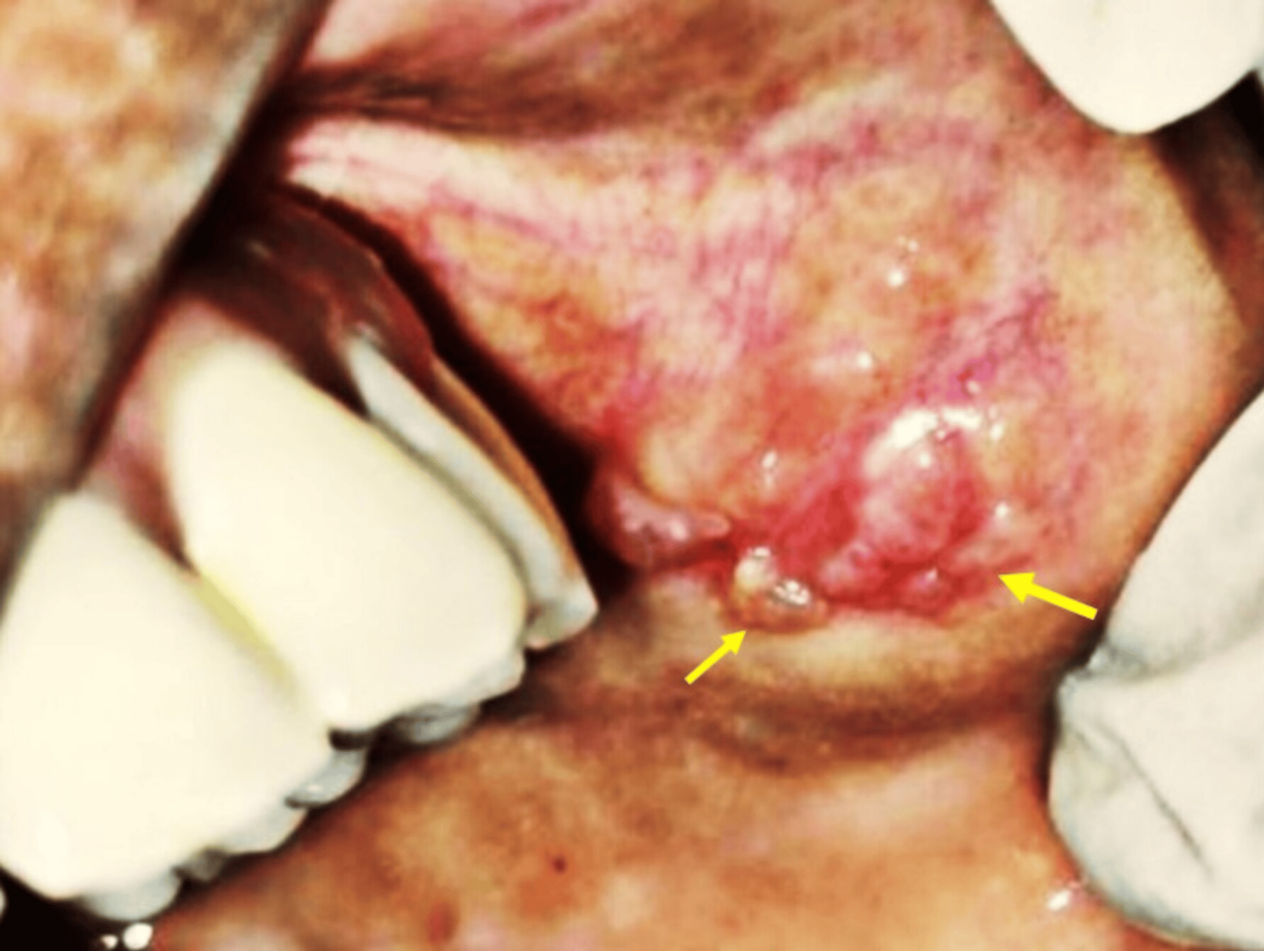
Intraoral clinical examination showing multiple discrete papules with a pebbly surface appearance (yellow arrows).

A 34-year-old woman reported to the Department of Oral Medicine and Radiology for a chief complaint of growth in the left side cheek region for the past one year. History revealed the lesion was initially smaller and attained the present size. Medical history revealed she was not a diabetic or hypertensive or had any other co-morbidities. A general examination revealed her vitals were stable. No similar growths are present on any other parts of her body. Intraoral clinical examination revealed multiple, well-circumscribed papules on her left buccal mucosa. The surface of the papules was shiny and stretched (Figure 1).

**Figure 2:**
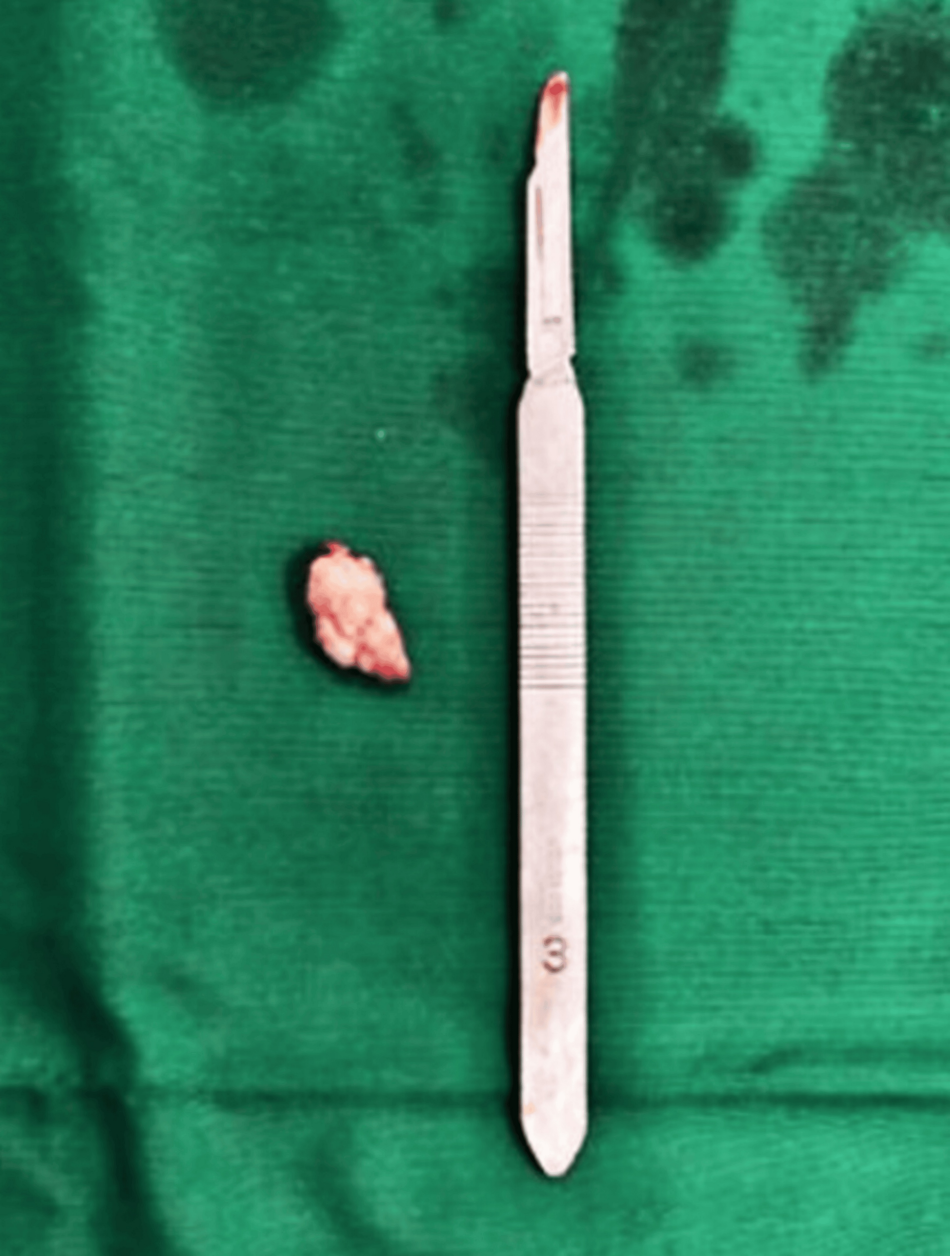
Excised specimen

On inspection, papules were seen on the left buccal mucosa, measuring about 1 x 1.5 cm in diameter, soft in consistency, and non-tender. The diascopy test was negative. The bleeding and clotting times were 9 and 12 minutes, respectively, and within biological ranges, without coagulopathies. The provisional diagnosis made was reactive hyperplasia on the buccal mucosa. The differential diagnoses made were inflammatory reactive hyperplasia and mucocele. Mucoceles usually appear solitary, rarely multiple. Excision biopsy of the lesion was performed under local anaesthesia using 2% lidocaine (Figure 2).

**Figure 3:**
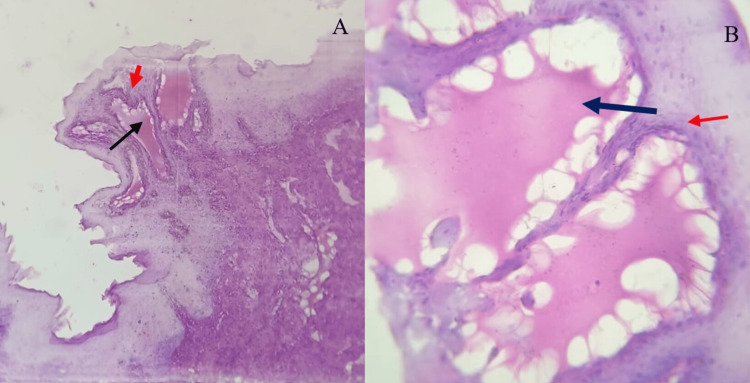
(A) Low-power (10x) and (B) high-power (40x) histopathological photomicrographs. The images reveal endothelial-lined vessels filled with lymph (black arrow) and overlying stratified squamous epithelium (red arrow).

The excised specimen was sent for histopathological examination. The histopathological examination revealed hyperplastic stratified squamous epithelium showing papillary folds. The subepithelial region showed numerous single endothelium-lined vessels of variable sizes and shapes filled with lymph (Figures 3A-3B).

**Figure 4:**
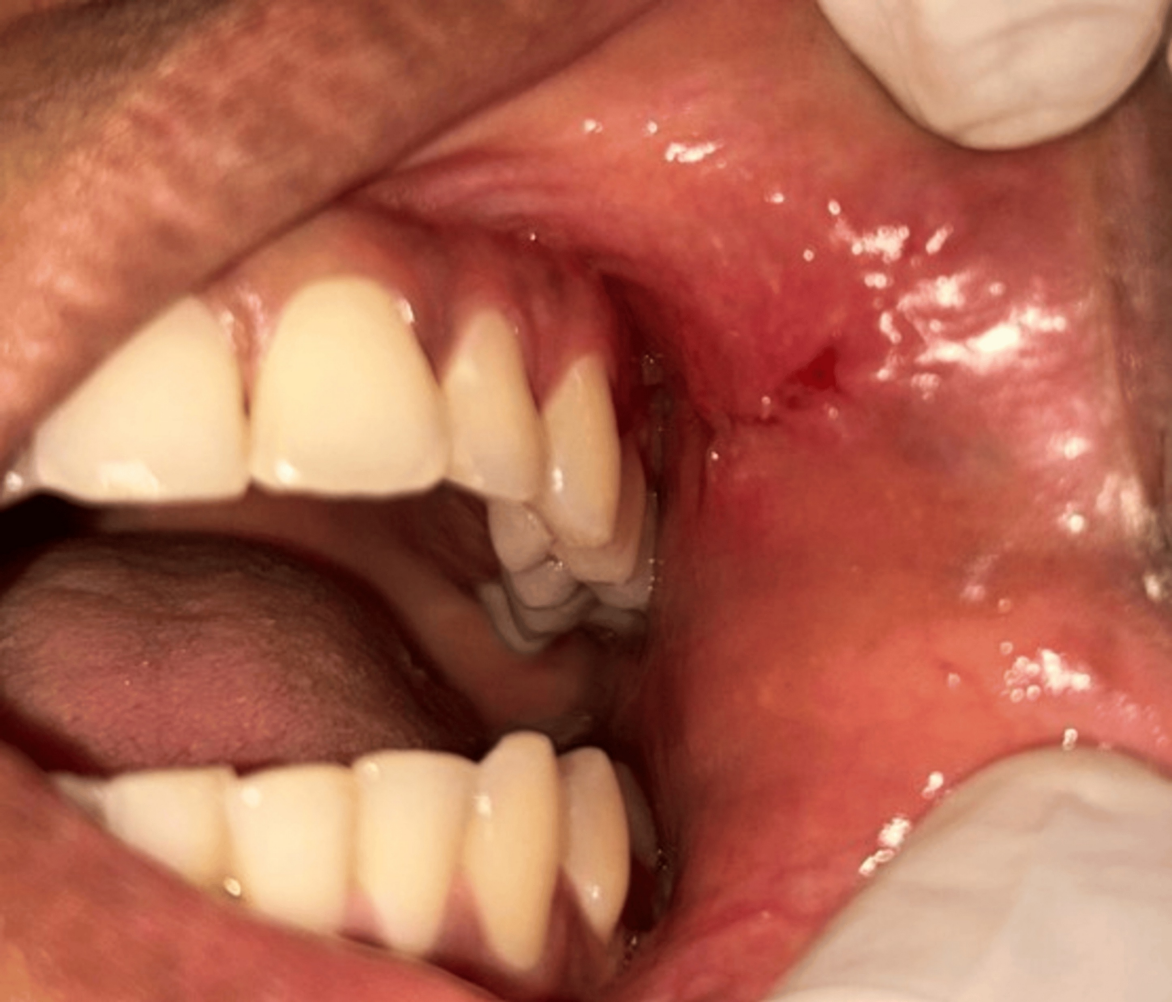
Follow-up clinical evaluation revealed satisfactory healing and no recurrence of the lesion.

Based on the clinical findings and histopathological features, a final diagnosis of cavernous LC was made. The patient was followed up after a week. Postoperative healing was satisfactory, and no lesion recurrence was reported (Figure 4).

## Discussion

Lymphangiomas or microcystic lymphatic malformations (MLM) are hamartomatous malformations containing ectatic lymphatic vessels [1]. Head and neck lymphangiomas account for about 75% of occurrences [1]. Sadasivan and Ramesh reported a mulberry-like appearance of lymphangioma in the attached gingiva [1]. Most cases (90%) occur in children under two years of age [1]. Most lymphatic anomalies affecting the oral cavity arise in the tongue, lips, palate, cheek and alveolar ridge [1]. Congenital lymphangiomas are linked to Turner, Edwards, Down, Noonan, and Patau syndromes [2]. LC occurs as transparent or hemorrhagic vesicular papules that resemble frog spawns or pebbles and contain clear fluid due to lymphatic fluid. Pruritus, discomfort, burning, lymphatic discharge, infection, and cosmetic issues may occur. The acquired form is usually observed in the axilla, inguinal, and vaginal areas with lymphoedema. Clinicians often misdiagnose them as cysts or lipomas. They appear soft, vary in size, and expand if not surgically removed. Turner syndrome, hydrops fetalis, and other congenital anomalies may be linked to posterior neck lesions [3].

Congenital or acquired lymphangiomas can be deep or superficial depending on the depth and size of the aberrant lymphatic veins. Cavernous lymphangiomas and cystic hygromas are well-defined congenital deep lymphangiomas. LC and acquired lymphangioma (lymphangiectasia) are superficial forms of lymphangioma [3,4]. Lymphangiomas occur in 1 per 2000-4000 live births. Male: female ratio was 2:1. Dermoscopy, biopsies, and imaging may be needed to confirm the diagnosis and assess the depth and extent of lymphangioma. Dermoscopic features include a "hypopyon-like" colour change from dark to light in some cavities [5].

The best treatment for any lymphangioma is surgical removal when possible. Since recurrence is common, lymphatic pathways must be extensively excised locally. Up to 81 months after surgical excision, LC recurrence rates were 23% [5]. Small, superficial lymphangiomas have better surgical outcomes such as in our case.

CO_2_ laser ablation, long-pulsed Nd:YAG laser, and electrosurgery therapies reduce symptoms. Cryotherapy, superficial radiation, and 23.4% hypertonic saline sclerotherapy are adjunct treatment modalities of lymphangiomatous malformations. Direct injection of 1% or 3% sodium tetradecyl sulphate, doxycycline, or ethanol can cause lymphatic abnormalities. Compression may reduce lymphoedema. Infection control is essential [6].

Palliative care for patients with lymphangioma includes sclerotherapy with hypertonic saline, electrofulguration or cautery, CO_2_ laser in continuous or pulsed modes, diode laser with radiofrequency current, pulsed dye laser [6,7]. The reactive hyperplasia results from sources of irritation such as a sharp broken carious tooth, friction from an orthodontic bracket, implant, sharp broken carious root stump, sharp interdental alveolar bone remaining after tooth extraction, sharp clasps or flanges or edges of ill-fitting dentures [9]. For children with head and neck lymphatic malformations, percutaneous doxycycline and Sotradecol treatment is safe and efficacious [10].

The most common consequences of LC are cellulitis and lymphatic fluid leakage. Oral sirolimus treatment serves as a first-line treatment for difficult lymphatic malformations in young people and an additional option for refractory lesions that do not respond to standard therapy [11]. For recurrent cheek lymphangioma, Achuthan et al. recommended a combined modality of sclerotherapy using an intralesional injection of bleomycin-guided under ultrasonography (USG) followed by surgery [12].

## Conclusions

LC is a rare and benign condition requiring a comprehensive diagnosis and treatment approach. With proper management, patients can experience significant improvement in their symptoms and quality of life. Timely diagnosis and intervention of lymphangiomas can prevent complications, such as infection, bleeding, and compression of surrounding tissues. A multidisciplinary team involving oral medicine specialists, oral maxillofacial surgeons and oral pathologists is essential in managing such lymphangiomas.
